# Comorbidity of Neurally Mediated Syncope and Allergic Disease in Children

**DOI:** 10.3389/fimmu.2020.01865

**Published:** 2020-08-28

**Authors:** Yaru Wang, Junbao Du, Hongfang Jin, Ying Liao

**Affiliations:** ^1^Department of Pediatrics, Peking University First Hospital, Beijing, China; ^2^Research Unit of Clinical Diagnosis and Treatment of Pediatric Syncope and Cardiovascular Diseases, Chinese Academy of Medical Sciences, Beijing, China

**Keywords:** comorbidity, neurally mediated syncope, allergic diseases, asthma, vasovagal syncope, postural tachycardia syndrome

## Abstract

Neurally mediated syncope (NMS) is the most common underlying disease of pediatric syncope, which generally includes vasovagal syncope (VVS), postural tachycardia syndrome (POTS), and situational syncope. Allergic diseases involving the respiratory system, digestive system, skin, and other systems are prevalent in children. In recent years, increasing attention has been paid to children with the comorbidity of NMS and allergic diseases. This article reviews the featured clinical manifestations and pathogenesis of the comorbidity according to the progress of related studies. Clinical studies have shown that the comorbidity rate of pediatric VVS and/or POTS with allergic diseases amounts to ~30–40%, referring to the whole population of children with VVS and/or POTS. Additionally, children with the comorbidity present some relatively special clinical characteristics. A series of mechanisms or regulatory factors relating to allergies, such as the imbalance of vasoactive elements, dysfunction of the autonomic nervous system (ANS), and autoimmunity may play a role in the development of the comorbidity. Moreover, 90% of children with cough syncope, a type of situational syncope, have a history of asthma, indicating a potential relationship between asthma and NMS. Further studies exploring the clinical characteristics and pathogenesis of the comorbidity are still needed to aid in the diagnosis and treatment of children with NMS.

## Introduction

Neurally mediated syncope (NMS), including vasovagal syncope (VVS), postural tachycardia syndrome (POTS), orthostatic hypertension (OHT), orthostatic hypotension (OH), situational syncope, and carotid sinus syndrome, is the most common underlying disease of pediatric syncope. VVS and POTS are the most common types of pediatric NMS (1, 2). In recent years, great attention has been paid to the comorbidities of pediatric NMS, such as migraine, mental illness, and chronic fatigue syndrome (3–5). Allergic diseases involving multiple systems, such as asthma (AS) and allergic rhinitis (AR) in the respiratory system and atopic dermatitis in skin, are prevalent in childhood. It is reported that 64.3% of adult AS patients also exhibited orthostatic dysregulation in Japan (6). A study in China showed that approximately 1/3 of the hospitalized children with VVS and/or POTS had a history of allergic diseases, and the percentage is as high as 42% for children with POTS alone (7). The study also indicated that allergic status might exacerbate the symptoms of pediatric NMS. Additionally, a review showed that 90% of pediatric cough syncope, which is a type of situational syncope, is related to AS (8). Although the pathogenesis of the comorbidity of pediatric NMS and allergic diseases is still unclear, several studies showed a potential role of regulatory factors (allergic inflammatory mediators, neuropeptides, and gasotransmitters), the regulation of autonomic nervous system and autoimmunity. We believe that further studies on the clinical characteristics and pathogenesis of pediatric NMS comorbid with allergic diseases might provide a new direction for the clinical diagnosis and treatment of NMS in children.

## Pediatric VVS and/or POTS Comorbid With Allergic Diseases

### Clinical Manifestations

VVS and POTS usually occur in older children and adolescents. Recurrent syncope is the major manifestation of VVS with predisposing factors, such as prolonged standing, quick changes from a supine or a squat to an upright position, emotional stress or fear, and a humid environment. POTS is a type of chronic orthostatic intolerance with predisposing factors similar to those of VVS. In addition to syncope, children with POTS usually suffer from dizziness, palpitation, chest distress, tremble, abdominal discomfort, or fatigue with long-term standing in daily life (9). VVS and POTS can be differentiated by a distinct response in a standing test or head-up tilt test in clinical practice. In some cases, VVS and POTS can coexist in the same young patient.

Clinical studies show that allergic diseases are the common comorbidity in children with VVS and POTS. A large cross-sectional study found that 20% of POTS patients were comorbid with AS (10). The proportion of pediatric VVS and/or POTS comorbid with allergic diseases, including AR, AS, food allergy, and atopic dermatitis, is up to 30–40% (7). AR is the most common comorbidity. The clinical manifestations of the comorbidity include older onset age of children, a shorter course of disease, and significantly increased eosinophils and IgE levels. In addition, the frequency of syncopal attacks in children with VVS comorbid with allergic diseases was much higher than that in pediatric VVS without allergic comorbidity (7).

Mast cell activation disorders (MCAD) are a group of conditions in which mast cells are either increased in amount, hyperreactive, or both. A portion of MCADs are secondary to allergic diseases (11–13). Several studies (14–17) show that POTS was a common comorbidity in young female patients with MCAD. Those patients present significant orthostatic tachycardia and hypertension, flushing, headache, dizziness, and gastrointestinal discomfort. It is also found that methylhistamine, leukotriene (LT), and prostaglandin D2 concentrations are significantly increased in the patients' urine. However, reports on the comorbidity of MCAD and POTS in children are still lacking.

### Pathogenesis

The differences in clinical characteristics between children with comorbidity of NMS and allergic diseases and those with NMS alone suggest that there may be certain particular mechanisms of pathogenesis in the comorbidity to be clarified. At present, it is believed that several mutual mechanisms, including the relative central hypovolemia, the dysregulation of peripheral vascular tone, and the imbalance of sympathetic and vagal functions, can all reduce the cardiac output when one stands upright and ultimately result in syncope in children with VVS and/or POTS (18, 19). Pathogenesis of allergic diseases may affect multiple aspects of orthostatic regulation to influence the features of children with the comorbidity of VVS/POTS and allergic diseases.

#### Vasoactive Factors Associated With Allergy

##### Inflammatory Mediators

**Figure 1:**
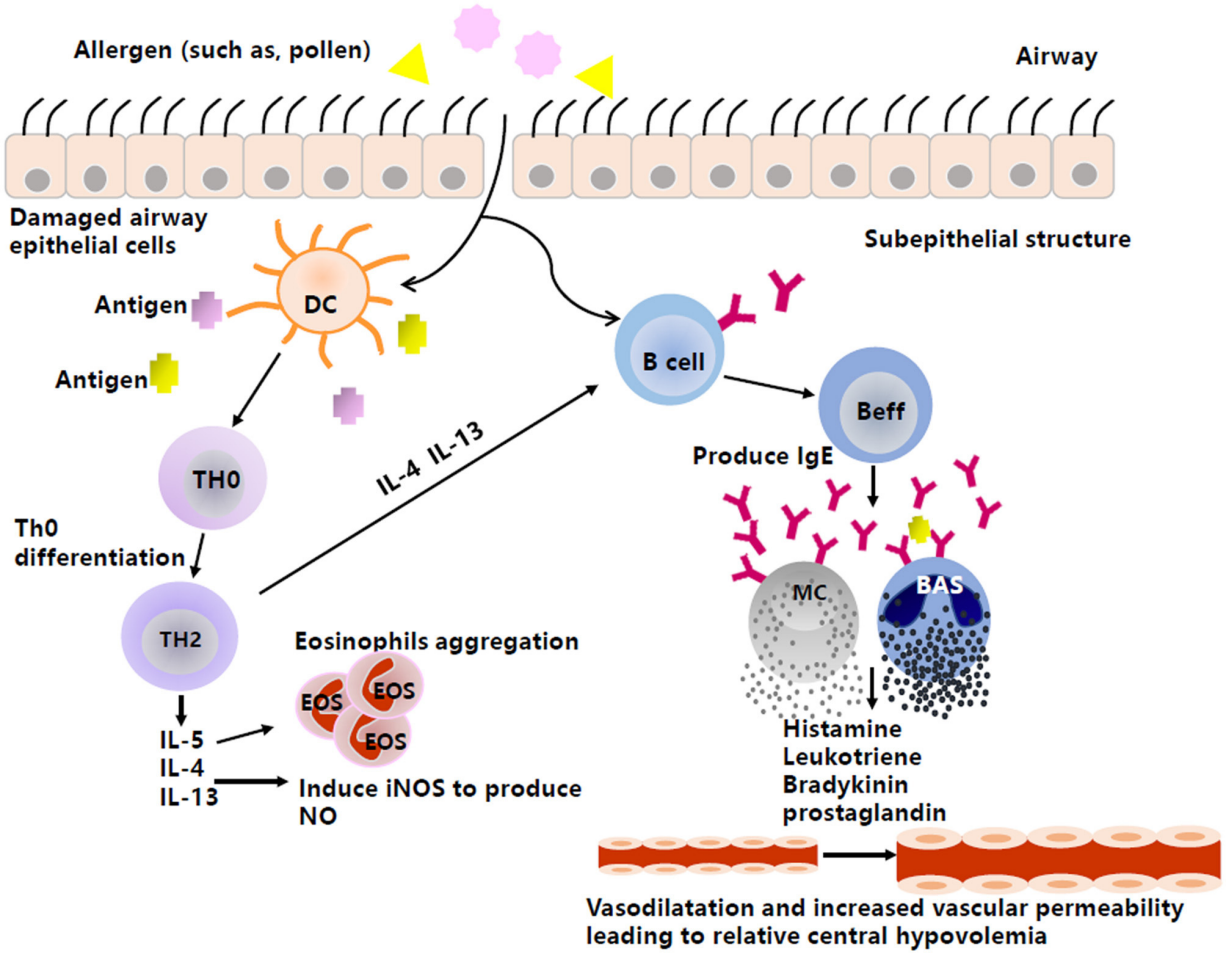
The production of vasoactive inflammatory mediators in type I anaphylaxis of airway. Inspiratory allergens, such as pollens, enter through the damaged airway epithelial cells. Then, DCs capture the allergens, present the antigens to Th0, and promote cell differentiation from Th0 to Th2. Th2 then produces cytokines, such as IL-4, IL-5, and IL-13. IL-5 can induce the aggregation of eosinophils, and IL-4 and IL-13 not only promote the production of NO by activating iNOS, but also stimulate Beff to produce IgE. The combination between antigen and antibody causes effector cells (e.g., MC, BAS) to produce and release histamine, leukotriene, etc., by degranulating, thus acting on peripheral blood vessels to mediate vasodilation and increase vascular permeability and resulting in relative central hypovolemia. DC, Dendritic cell; Th0, naive CD4+T cells; Th2, T cell type 2; Beff, Effector B cell; MC, Mast cell; BAS, Basophil; IL-4, Interleukin-4; IL-5, Interleukin-5; IL-13, Interleukin-13; iNOS, Inducible nitric oxide synthase; IgE, Immunoglobulin E.

AS and AR are generally IgE-mediated type I allergic diseases (Figure 1). When individuals are exposed to allergens, activated B cells differentiate into plasma cells and subsequently synthesize and secrete specific IgE (sIgE), which binds to high-affinity receptors on the surface of inflammatory cells, such as mast cells and basophils, resulting in a state of sensitization. When exposed to the same allergens again, sIgE antibodies can recognize the allergens and cause a cross-linking reaction, leading to the activation and degranulation of inflammatory cells to release a variety of inflammatory mediators, such as the classic histamine, LT, bradykinin, and prostaglandin, eventually leading to both acute and chronic inflammation, airway smooth muscle contraction, and increased secretion of mucus, etc. (20, 21).

Histamine, bradykinin, and prostaglandin are all known to have a diastolic effect on vascular smooth muscle and can enhance vascular permeability (22, 23). LT can also increase vascular permeability (23). These features may cause a reduction in venous return and inevitably result in relative central hypovolemia, which can be exacerbated by an upright position and facilitate orthostatic intolerance. However, whether these inflammatory mediators play a role in the mechanism of pediatric VVS and POTS still needs to be confirmed.

**Figure 2:**
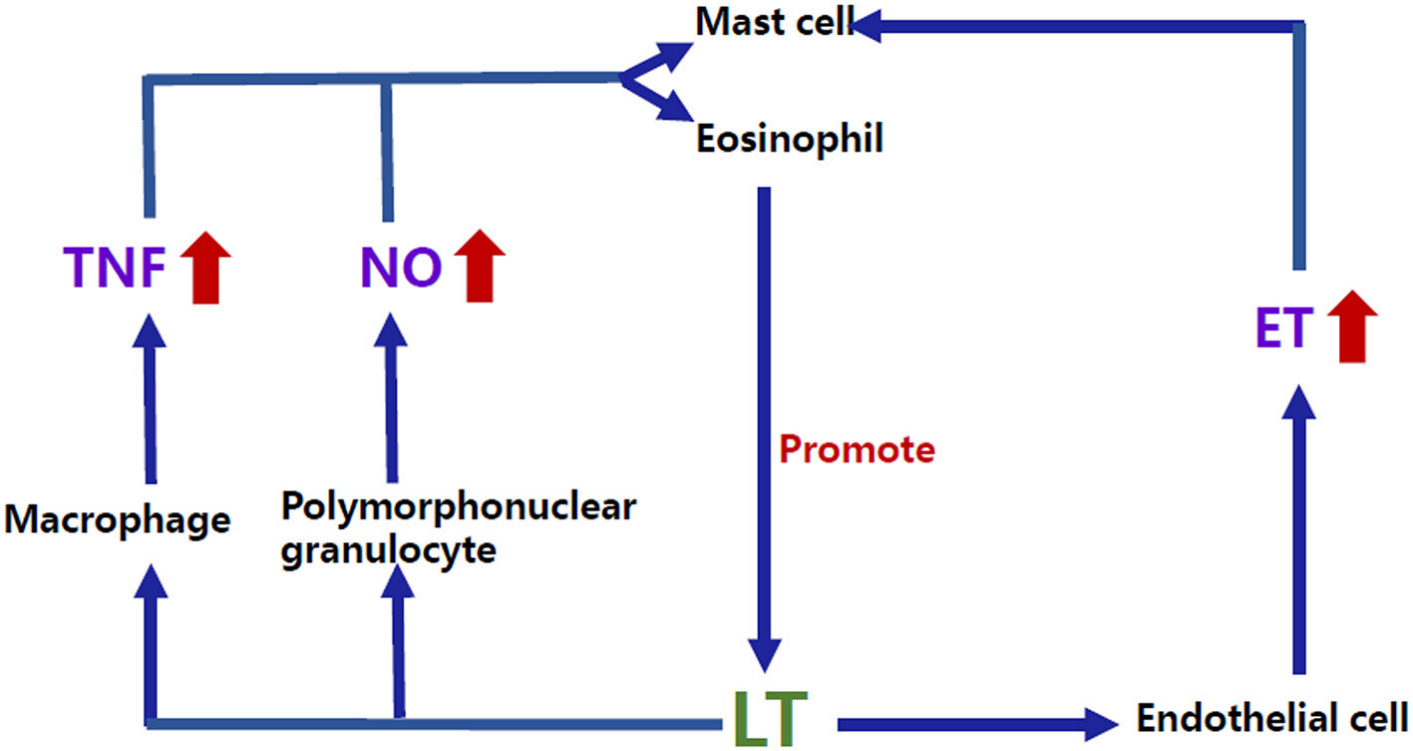
Bidirectional regulatory mechanisms between LT and other inflammatory mediators. ET-1 and TNF-α can induce the production of LTC_4_ by acting on the mast cells as well as eosinophils. Conversely, studies *in vitro* have shown that LTD_4_ can induce the production of TNF by stimulating the high-affinity receptor on macrophages, and LTC_4_ can also regulate the production of ET-1. NO can increase the production of LT through human mast cells, and LTB_4_, LTC_4_, and LTD_4_ can also promote NO release by activating the polymorphonuclear granulocyte surface receptors. LTB_4_, Leukotriene B_4_; LTC_4_, Leukotriene C_4_; LTD_4_, Leukotriene D_4_; LT, Leukotriene; ET, Endothelin; TNF, Tumor necrosis factor; NO, Nitric oxide.

In addition to the direct role of these classic mediators, the complex interactions among the allergy-related inflammatory mediators may provide some clues for the comorbidity. For example, LT, one of the essential factors in the allergic response, can interact with other factors, such as endothelin (ET) and tumor necrosis factor α (TNF-α) (24) (Figure 2). Studies have shown that ET-1 (25) and TNF-α (26) can induce the production of leukotriene C_4_ (LTC_4_) by acting on mast cells as well as eosinophils. Conversely, studies *in vitro* have shown that leukotriene D_4_ (LTD_4_) can induce the production of TNF by stimulating the high-affinity receptor on alveolar macrophages (27) and that LTC_4_ can also regulate the production of ET-1 (28). Interestingly, previous studies have shown that plasma ET levels were increased in children with VVS (29), suggesting an imbalance of vascular tone regulators in children with NMS. Similar to the findings in NMS children, plasma ET-1 levels in children with AS significantly increase in the acute attack stage compared with the remission stage (30). ET-1 is also elevated in the airway epithelium and involved in bronchoconstriction and airway remodeling in patients with AS (31, 32), indicating that ET may be a candidate factor related to the pathogenesis of the comorbidity of NMS and AS in children. For TNF, Gallegos (33) found that soluble tumor necrosis factor receptor 1 (sTNFR1) was detected in the blood of pediatric patients with VVS. Under the influence of predisposing factors, such as prolonged standing and postural changes, sTNFR1 was suddenly reduced, and its inhibitory effect on TNF was weakened, subsequently increasing the secretion of prostaglandin E_2_ and nitric oxide (NO) and leading to vasodilation and syncopal attacks. Nevertheless, the role of TNF in the pathogenesis of comorbidity needs further exploration in the future.

##### Neuropeptides

Other vasoactive factors are involved in AS and can cause vasodilation and enhance microvascular permeability. When the sensory nerve endings are exposed to antigens or inflammatory mediators due to damage of the top covered airway epithelium, they can reversibly release a group of neuropeptides, such as neurokinin A, calcitonin-related peptides, substance P, bradykinin, tachykinin, and neuropeptide Y, which may lead to bronchial constriction, microvascular leakage, and mucus hypersecretion (34–36). Among them, calcitonin-related peptides, substance P, bradykinin, and tachykinin all have the effect of vasodilatation; in contrast, neuropeptide Y exerts the effect of vasoconstriction. It was reported that the plasma neuropeptide Y level was significantly decreased (37) and substance P was increased (38) in children with VVS in the supine position. It is worth further exploring whether these vasoactive neuropeptides are involved in the pathogenesis of the comorbidity.

##### Nitric Oxide

NO is an active gasotransmitter that is released when L-arginine transforms into L-citrulline by oxidation under the catalytic action of nitric oxide synthase (NOS) (39). NO can pass through biofilms by free diffusion and play an extensive regulatory role in the nervous, immune, and cardiovascular systems. NO is reported to be involved in the pathogenesis of both pediatric AS and NMS (40–42). In the pathogenesis of AS, exposure to allergens induces helper T cell type 2 (Th2) to produce a series of inflammatory mediators, such as interleukin-4 (IL-4), interleukin-5 (IL-5), and interleukin-13 (IL-13). IL-5 promotes the aggregation of eosinophils (43), and IL-4 and IL-13 trigger downstream signaling and upregulate the synthesis of inducible nitric oxide synthase (iNOS) mRNA, leading to a significant increase in the levels of fractional exhaled nitric oxide (FeNO) (44). iNOS can be detected in the airway epithelial cells of patients with AS (45), and the levels of FeNO in AS are significantly increased (42). Therefore, it is supposed that NO plays an important role in the pathogenesis of AS. A guideline on pediatric pulmonary function and airway nontraumatic inflammation indicators suggests that FeNO is an airway inflammation indicator and a noninvasive indicator that can be used to measure the levels of eosinophilic inflammation in children with AS (46). There is also evidence for interactions between NO and LT (Figure 2), which may be another way that NO participates in the pathogenesis of allergic diseases. For example, NO (47) can increase the production of LT through human mast cells, and leukotriene B_4_ (LTB_4_), LTC_4_, and LTD_4_ can promote NO release by activating polymorphonuclear granulocyte surface receptors (48). Studies about the role of NO in pediatric NMS have focused on vascular endothelium-derived NO. Previous studies have shown that children with both POTS and VVS had increased plasma NO levels and significantly enhanced flow-mediated vasodilation (FMD) (40, 41, 49). Moreover, NOS activity was enhanced and was proportional to FMD in children with POTS (40). Genotype analysis of the NOS gene also revealed that greater endothelial NOS activity may be associated with the pathogenesis of POTS (50). These results indicated abnormal vascular endothelial function in children with VVS and POTS, which may result in enhanced vasodilatation and peripheral blood pooling when upright, exacerbating orthostatic intolerance. Another study explored the vascular endothelial function as well as the arterial stiffness using the reactive hyperemia index (RHI) and augmentation index (AIx). However, there was no significant difference in vascular endothelial function between AS and the control group although poorer arterial elasticity was found in the AS group (51). In another study, it was shown that FMD was decreased in children with AS and that reduced FMD was correlated with the decreased forced expiratory volume in 1 second (FEV_1_) and the function of the small airway, suggesting that vascular endothelial dysfunction exists in children with AS (52). Because NO plays a significant role in the pathogenesis of both pediatric NMS and AS, it is worth researching FeNO as well as circulatory NO levels in children with allergic diseases comorbid with NMS to find the possible influence of NO in the comorbidity.

Dysregulation of peripheral vascular tone and relative central hypovolemia are important pathogenesis mechanisms that cannot be ignored in children with NMS. Therefore, whether the known allergy-related vasoactive factors contribute to the relative central hypovolemia by enhancing vasodilation and/or increasing vascular permeability in children with the comorbidity of NMS and allergic diseases remains to be further studied. Additionally, the mechanisms for mutual regulation among each factor are not fully understood.

#### Dysfunction of the Autonomic Nervous System

The imbalance of sympathetic and vagal functions is one of the important mechanisms for NMS. Although AS is defined as a heterogeneous disease characterized by chronic airway inflammation and airway hyper-responsiveness, some studies also support that AS is associated with dysfunction of the autonomic nervous system (ANS). It is believed that patients with AS have increased parasympathetic reactivity, which may be related to the severity of the disease or the long-term use of medicine, such as β2 adrenergic agonists and anticholinergic medicines (53, 54). Whether this dysfunction of the ANS in patients with AS accounts for a tendency of NMS remains to be further studied.

#### Autoimmunity

Autoimmunity is another mechanism that is involved in both AS and NMS. On the one hand, it is believed that autoimmune phenomenon is found in patients with AS (55). Kero reports that the cumulative incidence of AS in children with celiac disease or rheumatoid arthritis is significantly higher (10.0%) than that in children without these autoimmune diseases (3.4%) (56). Previous studies also demonstrate circulating autoantibodies against β2 adrenergic receptor, epithelial antigen, and nuclear antigen in AS patients (55). Although the existence of these circulating autoantibodies may just be a concomitant phenomenon of chronic inflammation in AS rather than the major pathogenic factor, these autoantibodies may be a potential factor affecting the function of the ANS. On the other hand, increasing evidence has shown that POTS is related to autoimmunity (57). Blitshteyn found that one quarter of the patients had antinuclear antibodies (ANA), almost one third of the patients had autoimmune markers, and one fifth of the patients had coexisting autoimmune diseases (58). In addition, G-protein-coupled adrenergic autoantibodies, muscarinic autoantibodies, and angiotensin II type 1 receptor autoantibodies are all elevated in POTS patients (59, 60). It is not clear whether autoimmune mechanisms are involved in the occurrence of comorbidity.

## Situational Syncope Comorbid with Allergic Diseases in Children

### Clinical Manifestations

Cough syncope is a form of situational syncope characterized by paroxysmal coughing, facial congestion or cyanosis, and loss of consciousness, and the episode of cough syncope usually occurs within seconds and is followed by recovery within seconds to minutes (61, 62). Cough syncope in children is thought to be associated with AS. A recent review showed that 90.3% of children with cough syncope had a history of AS (8). As early as 1876, Charcot first described the loss of consciousness after coughing in children (63). Nearly a century later, Robert described twelve children with cough syncope companies with AS, of whom eleven were allergic to inhalant allergens and six developed allergic symptoms to certain foods; pulmonary function was measured in eight children, who all showed reversible airflow limitation (64).

### Pathogenesis

The exact mechanisms for cough syncope are not fully understood. In the past, cough followed by a transient loss of consciousness (TLOC) used to be considered a kind of epileptic seizure (65), and then an increasing number of researchers believed that changes in the circulatory system might be the real cause of the TLOC. In 1984, DeMaria (66) confirmed that cough syncope was not an epileptic seizure, as there was no discharge in the electroencephalogram during the episodes of cough syncope in adult patients.

#### Changes in Thoracic Compliance

One of the hypotheses put forward to explain the complex pathogenesis of cough syncope is related to the change in thoracic compliance. In general, it is speculated that elevated intrathoracic pressures are needed to trigger an attack of cough syncope, and the cough caused by AS is a potential trigger. Most reported cases of pediatric cough syncope are related to AS. One apparent fact is that cough is a common clinical manifestation in children with AS. Furthermore, Katz's study suggests that the decreased thoracic and pulmonary compliance in patients with AS may predispose them to have cough syncope (61). Subsequently, the explanation of the mechanisms for cough syncope focused on the increased chest and abdominal pressures caused by cough. It is believed that, during cough syncope, the increased intrathoracic pressure induced by coughing and enhanced by decreased compliance of the thoracic wall diminishes the cardiac output, consequently leading to decreased systemic blood pressure as well as insufficient cerebral perfusion. In addition, the cerebral blood vessels are thought to be compressed because of an increased extravascular pressure produced by the elevated intracranial pressure when coughing, further diminishing the cerebral perfusion. In some cases, a concussion-like effect may be caused by the rapid increasing cerebrospinal fluid pressure (8, 67). All of the above changes occur in the course of cough syncope.

#### Autonomic Nervous Reflex

It has been suggested that diminished cerebral blood flow due to vagal excitation may be the main mechanism for cough syncope (68). Early in 1953, baroreflex was thought to be related to cough syncope (69). However, the role of baroreflex in the pathogenesis of cough syncope had been confirmed by Benditt et al. until 2005 (70). Their findings showed that patients with cough syncope presented more severe hypotension over an even longer period than those with other causes of syncope and that the positive chronotropic response was usually suppressed in patients with cough-triggered hypotension, indicating a more significant vagal excitatory response. These results support that cough syncope is characterized by a cough-triggered neurally mediated reflex causing hypotension and/or bradycardia.

## Summary

**Figure 3:**
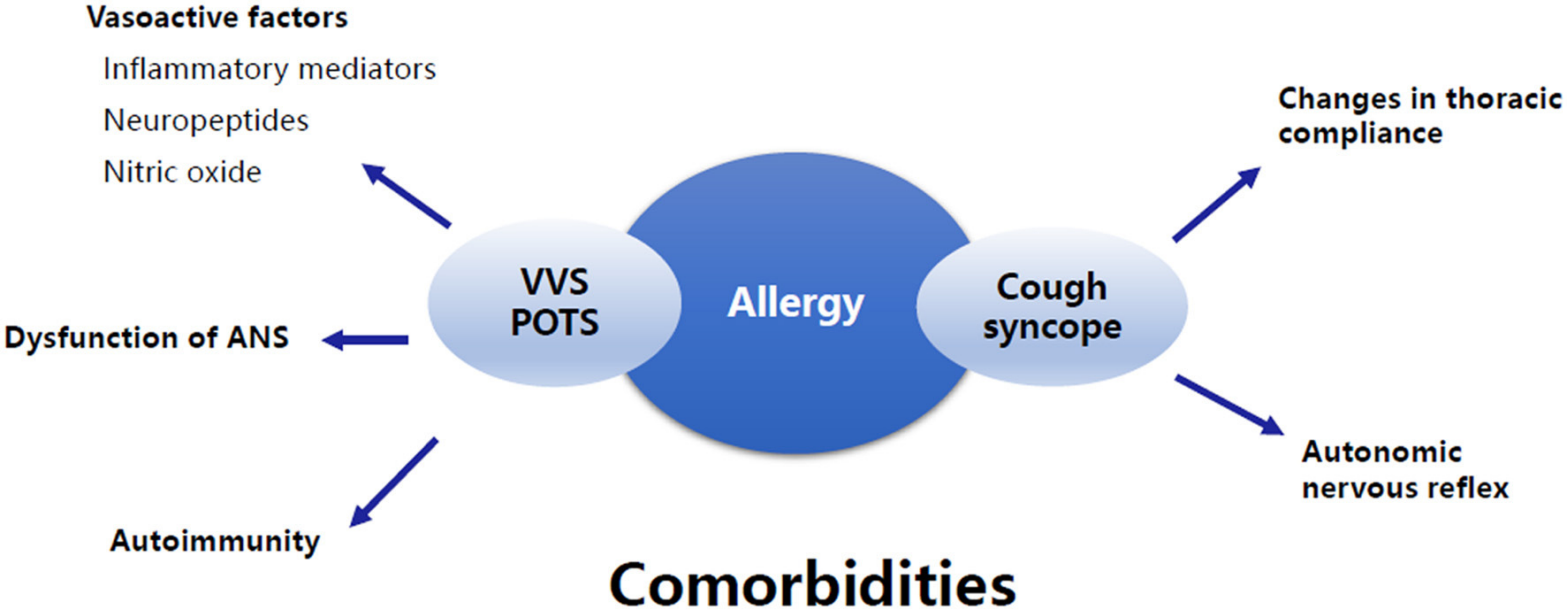
Summary of the underlying mechanisms of the comorbidity of allergic diseases and NMS. The underlying mechanisms of the comorbidity of allergic diseases and VVS and/or POTS involve vasoactive factors associated with allergy, dysfunction of ANS, and autoimmunity. The comorbidity of allergic diseases and cough syncope is supposed to be related to changes in thoracic compliance and autonomic nervous reflex. VVS, Vasovagal syncope; POTS, Postural tachycardia syndrome; ANS, Autonomic nervous system.

Increasing evidence suggests that allergic diseases are common comorbidities of pediatric NMS, but the data are limited, and the pathogenesis is unclear. It can be speculated that the disturbance of allergy-related vasoactive mediators, autonomic nervous dysfunction, autoimmunity, changes in thoracic compliance, and autonomic nervous reflex may be potential mechanisms for the comorbidity (Figure 3). Further studies are needed to confirm the exact mechanisms. In addition, there are several other entities of NMS, such as OH, OHT, and other forms of situational syncope, that may share some common pathophysiological mechanisms with VVS, POTS, and cough syncope as mentioned above. However, no studies have been published on the comorbidity of these types of NMS and allergic diseases in children. We believe that great attention should be paid to this topic in the future because comprehensive studies on the clinical characteristics and pathogenesis will help to improve the understanding and therapeutic efficacy of pediatric NMS comorbid with allergic diseases.

## Author Contributions

YW wrote the review. JD and HJ supervised the program. YL revised and edited the manuscript. All authors contributed to the article and approved the submitted version.

## Conflict of Interest

The authors declare that the research was conducted in the absence of any commercial or financial relationships that could be construed as a potential conflict of interest.

## References

[1] Chinese Medicine Subspecialty of Cardiovascular Study Group Children syncope diagnostic guidelines. Chin J Pediatr. (2016) 54:246–50. 10.3760/cma.j.issn.0578-1310.2016.04.003

[2] Wang C Li Y Liao Y Tian H Huang M Dong XY 2018 Chinese Pediatric Cardiology Society (CPCS) guideline for diagnosis and treatment of syncope in children and adolescents. Sci Bull. (2018) 63:1558–64. 10.1016/j.scib.2018.09.019 36751076

[3] Vallejo M Martinez-Martinez LA Grijalva-Quijada S Olguín-Ruvalcaba HM Salas E Hermosillo AG. Frequency of migraine in patients with vasovagal syncope. Int J Cardiol. (2014) 171:e14–5. 10.1016/j.ijcard.2013.11.132 24360080

[4] Zysko D Melander O Fedorowski A. Vasovagal syncope related to emotional stress predicts coronary events in later life. Pacing Clin Electrophysiol. (2013) 36:1000–6. 10.1111/pace.12138 23614671

[5] Reynolds GK Lewis DP Richardson AM Lidbury BA. Comorbidity of postural orthostatic tachycardia syndrome and chronic fatigue syndrome in an Australian cohort. J Intern Med. (2014) 275:409–17. 10.1111/joim.12161 24206536

[6] Sakuramoto M Himeno T Minoguchi K Watanabe N Kobayashi H Koseki T. The prevalence of orthostatic dysregulation complicated with bronchial asthma. Arerugi. (1997) 46:1123–31. 9436329

[7] Liao Y Zhang Q Li H Wang Y Liu P Du J. Co-morbidity of vasovagal syncope and postural tachycardia syndrome with allergic diseases in children. J Peking Univ Health Sci. (2017) 49:41–6. 10.3969/j.issn.1671-167X.2017.05.007 29045956

[8] Dicpinigaitis PV Lim L Farmakidis C. Cough syncope. Respir Med. (2014) 108:244–51. 10.1016/j.rmed.2013.10.020 24238768

[9] Wang Y Du J Jin H Differential diagnosis of vasovagal syncope and postural tachycardia syndrome in children. World J Pediatr. (2020) 10.1007/s12519-019-00333-4. [Epub ahead of print]. 32020440

[10] Shaw BH Stiles LE Bourne K Green EA Shibao CA Okamoto LE. The face of postural tachycardia syndrome-insights from a large cross-sectional online community-based survey. J Intern Med. (2019) 286:438–48. 10.1111/joim.12895 30861229 PMC6790699

[11] Giannetti MP Akin C Castells M. Idiopathic anaphylaxis: a form of mast cell activation syndrome. J Allergy Clin Immunol Pract. (2020) 8:1196–201. 10.1016/j.jaip.2019.10.048 32276688

[12] Akin C Valent P Metcalfe DD. Mast cell activation syndrome: proposed diagnostic criteria. J Allergy Clin Immunol. (2010) 126:1099–104.e4. 10.1016/j.jaci.2010.08.035 21035176 PMC3753019

[13] Hamilton MJ Hornick JL Akin C Castells MC Greenberger NJ. Mast cell activation syndrome: a newly recognized disorder with systemic clinical manifestations. J Allergy Clin Immunol. (2011) 128:147–52. 10.1016/j.jaci.2011.04.037 21621255

[14] Shibao C Arzubiaga C Roberts LJ Raj S Black B Harris P. Hyperadrenergic postural tachycardia syndrome in mast cell activation disorders. Hypertension. (2005) 45:385–90. 10.1161/01.HYP.0000158259.68614.40 15710782

[15] Emily M Jorge E Satish R. Postural tachycardia syndrome: beyond orthostatic intolerance. Curr Neurol Neurosci Rep. (2015) 15:60. 10.1007/s11910-015-0583-8 26198889 PMC4664448

[16] Bonamichi-Santos R Yoshimi-Kanamori K Giavina-Bianchi P Aun MV. Association of postural tachycardia syndrome and Ehlers-Danlos Syndrome with mast cell activation disorders. Immunol Allergy Clin North Am. (2018) 38:497–504. 10.1016/j.iac.2018.04.004 30007466

[17] Raj SR. Postural tachycardia syndrome (POTS). Circulation. (2013) 127:2336–42. 10.1161/CIRCULATIONAHA.112.144501 23753844 PMC3756553

[18] Song J Wang Y Li H Du J Research progress of mechanisms for vasovagal syncope in children. Chin J Appl Clin Pediatr. (2018) 33:478–80. 10.3760/cma.j.issn.2095-428X.2018.06.020

[19] Zheng X Chen Y Du J. Recent advances in the understanding of the mechanisms underlying postural tachy-cardia syndrome in children: practical implications for treatment. Cardiol Young. (2017) 27:413–7. 10.1017/S1047951116002559 27938459

[20] Barnes PJ Asthma mechanisms. Med. (2016) 44:265–70. 10.1016/j.mpmed.2016.02.020

[21] Akdis CA Global Atlas of Allergy. Zurich: European Academy of Allergy and Clinical Immunology (2014) 39–42.

[22] Ashina K Tsubosaka Y Nakamura T Omori K Kobayashi K Hori M. Histamine induces vascular hyperpermeability by increasing blood flow and endothelial barrier disruption *in vivo*. PLoS ONE. (2015) 10:e0132367. 10.1371/journal.pone.0132367 26158531 PMC4497677

[23] Hart P. Regulation of the inflammatory response in asthma by mast cell products. Immunol Cell Biol. (2001) 79:149–53. 10.1046/j.1440-1711.2001.00983.x 11264709

[24] Peters-Golden M Gleason MM Togias A. Cysteinyl leukotrienes multi-functional mediators in allergic rhinitis. Clin Exp Allergy. (2006) 36:689–703. 10.1111/j.1365-2222.2006.02498.x 16776669 PMC1569601

[25] Yamamura H. Endothelin-1 induces release of histamine and leukotriene C4 from mouse bone marrow-derived mast cells. Eur J Pharmacol. (1994) 257:235–42. 10.1016/0014-2999(94)90134-1 7522171

[26] Takafuji S Bischoff SC De Weck AL Dahinden CA. Opposing effects of tumor necrosis factor-alpha and nerve growth factor upon leukotriene C4 production by human eosinophils triggered with N-formyl-methionyl-leucyl-phenylalanine. Eur J Immunol. (1992) 22:969–74. 10.1002/eji.1830220414 1551409

[27] Menard G Bissonnette EY. Priming of alveolar macrophages by leukotriene D(4): potentiation of inflammation. Am J Respir Cell Mol Biol. (2000) 23:572–7. 10.1165/ajrcmb.23.4.4152 11017925

[28] Patrignani P Modica R Bertolero F Patrono C. Differential effects of leukotriene C4 on endothelin-1 and prostacyclin release by cultured vascular cells. Pharmacol Res. (1993) 27:281–5. 10.1006/phrs.1993.1027 8327407

[29] Magerkurth C Riedel A Braune S. Permanent increase in endothelin serum levels in vasovagal syncope. Clin Auton Res. (2005) 15:299–301. 10.1007/s10286-005-0291-6 16032385

[30] EL-Gamal Y Hossny E Awwad K Mabrouk R Boseila N. Plasma endothelin-1 immunoreactivity in asthmatic children. Ann Allergy Asthma Immunol. (2002) 88:370–3. 10.1016/S1081-1206(10)62366-6 11991554

[31] Chalmers GW MacLeod KJ Thomson LJ Little SA Patel KR McSharry C. Sputum cellular and cytokine responses to inhaled endothelin-1 in asthma. Clin Exp Allergy. (1999) 29:1526–31. 10.1046/j.1365-2222.1999.00496.x 10520081

[32] Carratu P Scuri M Stybio JI Wanner A Glassberg MK. ET-1 induces mitogenesis in ovine airway smooth muscle cells via ETA and ETB receptors. Am J Physiol. (1997) 272:L1021–4. 10.1152/ajplung.1997.272.5.L1021 9176269

[33] Gallegos A Marquez-Velasco R Allende R Gómez-Flores JR Cázares-Campos I González-Hermosillo A. Serum concentrations of nitric oxide and soluble tumor necrosis factor receptor 1(sTNFR1) in vasovagal syncope: effect of orthostatic challenge. Int J Cardiol. (2013) 167:2321–2. 10.1016/j.ijcard.2012.11.019 23182003

[34] Barnes PJ. Neural mechanisms in asthma. Br Med Bull. (1992) 48:149–68. 10.1093/oxfordjournals.bmb.a072531 1352167

[35] Ricciardolo FL Rado V Fabbri LM Sterk PJ Di Maria GU Geppetti P. Bronchocon-striction induced by citric acid inhalation in guinea pigs role of tachykinins, bradykinin, and nitric oxide. Am J Respir Crit Care Med. (1999) 159:557–62. 10.1164/ajrccm.159.2.9804022 9927373

[36] Barnes PJ. Neuropeptides and asthma. Eur J Pharmacol. (1991) 143:S28–32. 10.1164/ajrccm/143.3_Pt_2.S28 1706152

[37] Liao Y Xu WR Li HX Tang CS Jin HF Du JB. Plasma neuropeptide Y levels in vasovagal syncope in children. Chin Med J. (2017) 130:2778–84. 10.4103/0366-6999.219157 29176136 PMC5717855

[38] Ellenbogen KA Morillo CA Wood MA Gilligan DM Eckberg DL Smith ML. Neural monitoring of vasovagal syncope. Pacing Clin Electrophysiol. (1997) 20:788–94. 10.1111/j.1540-8159.1997.tb03905.x 9080511

[39] Welch G Loscalzo J. Nitric oxide and the cardiovascular system. J Card Surg. (1994) 9:361–71. 10.1111/j.1540-8191.1994.tb00857.x 8054733

[40] Liao Y Chen S Liu X Zhang Q Ai Y Wang Y. Flow-mediated vasodilation and endothelium function in children with postural orthostatic tachycardia syndrome. Am J Cardiol. (2010) 106:378–82. 10.1016/j.amjcard.2010.03.034 20643249

[41] Du J Chen SQ Zhu HF Changes of NO, 5-HT and SpO2 in children with vasovagal syncope. Med J West China. (2012) 24:552–4. 10.3969/j.issn.1672-3511.2012.03.048

[42] Guo FH Comhair SA Zheng S Dweik RA Eissa NT Thomassen MJ. Molecular mechanisms of increased nitric oxide (NO) in asthma: evidence for transcriptional and post-translational regulation of NO synthesis. J Immunol. (2000) 164:5970–80. 10.4049/jimmunol.164.11.5970 10820280

[43] Johansso MW Khanna M Bortnov V Annis DS Nguyen CL Mosher DF. IL-5-stimulated eosinophils adherent to periostin undergo stereotypic morphological changes and ADAM8-dependent migration. Clin Exp Allergy. (2017) 47:1263–74. 10.1111/cea.12934 28378503 PMC5623171

[44] Yamamoto M Tochino Y Chibana K Trudeau JB Holguin F Wenzel SE. Nitric oxide and related enzymes in asthma: relation to severity, enzyme function and inflammation. Clin Exp Allergy. (2012) 42:760–8. 10.1111/j.1365-2222.2011.03860.x 22092728 PMC3650251

[45] Redington AE Meng QH Springall DR Evans TJ Créminon C Maclouf J. Increased expression of inducible nitric oxide synthase and cyclo-oxygenase-2 in the airway epithelium of asthmatic subjects and regulation by corticosteroid treatment. Thorax. (2001) 56:351–7. 10.1136/thorax.56.5.351 11312402 PMC1746058

[46] Chinese Medical Association Pediatrics Society Respiratory Group Lung Function Cooperation Group, Editorial Board of Chinese Practical Clinical Journal of Pediatrics Series of guidelines for indicators of pulmonary function and airway non-traumatic inflammation in children (seven): monitoring of the exhaled gas nitric oxide. Chin J Appl Clin Pediatr. (2017) 32:1622–7. 10.3760/cma.j.issn.2095-428X.2017.21.006

[47] Gilchrist M McCauley SD Befus AD. Expression, localization, and regulation of NOS in human mast cell lines: effects on leukotriene production. Blood. (2004) 104:462–9. 10.1182/blood-2003-08-2990 15044250

[48] Larfars G Lantoine F Devynck MA Palmblad J Gyllenhammar H. Activation of nitric oxide release and oxidative metabolism by leukotrienes B4, C4, and D4 in human polymorphonuclear leukocytes. Blood. (1999) 93:1399–405. 10.1182/blood.V93.4.1399 9949184

[49] Zhang Q Du J Li Y Ai Y Endothelial function in children with vasovagal syncope via color doppler flow imaging. Chin J Prac Pediatr. (2005) 20:482–4. 10.3969/j.issn.1005-2224.2005.08.013

[50] Garland EM Winker R Williams SM Jiang L Stanton K Byrne DW. Endothelial NO synthase polymorphisms and postural tachycardia syndrome. Hypertension. (2005) 46:1103–10. 10.1161/01.HYP.0000185462.08685.da 16203873

[51] Augusto LS Silva GC Pinho JF Aires RD Lemos VS Ramalho LF. Vascular function in asthmatic children and adolescents. Respir Res. (2017) 18:17. 10.1186/s12931-016-0488-3 28095859 PMC5240276

[52] Butov D Makieieva N Vasylchenko Y Biriukova M Serhiienko K Morozov O. Endothelial dysfunction in children with clinically stable and exacerbated asthma. Adv Respir Med. (2019) 87:7–13. 10.5603/ARM.a2019.0002 30830954

[53] Lemanske RF Kaliner MA. Autonomic nervous system abnormalities and asthma. Am Rev Respir Dis. (1990) 141:S157–61. 10.1164/ajrccm/141.3_Pt_2.S157 2155565

[54] Jartti T. Asthma, asthma medication and autonomic nervous system dysfunction. Clin Physiol. (2001) 21:260–9. 10.1046/j.1365-2281.2001.00323.x 11318835

[55] Mukherjee M Nair P. Autoimmune responses in severe asthma. Allergy Asthma Immunol Res. (2018) 10:428–47. 10.4168/aair.2018.10.5.428 30088364 PMC6082822

[56] Kero J Gissler M Hemminki E Isolauri E Could th1 and th2 diseases coexist? Evaluation of asthma incidence in children with coeliac disease, type 1 diabetes, or rheumatoid arthritis: a register study. J Allergy Clin Immunol. (2001) 108:781–3. 10.1067/mai.2001.119557 11692104

[57] Dahan S Tomljenovic L Shoenfeld Y. Postural orthostatic tachycardia syndrome (POTS)-a novel member of the autoimmune family. Lupus. (2016) 25:339–42. 10.1177/0961203316629558 26846691

[58] Blitshteyn S. Autoimmune markers and autoimmune disorders in patients with postural tachycardia syndrome (POTS). Lupus. (2015) 24:1364–9. 10.1177/0961203315587566 26038344

[59] Gunning WT Kvale H Kramer PM Karabin BL Grubb BP. Postural orthostatic tachycardia syndrome is associated with elevated G-protein coupled receptor autoantibodies. J Am Heart Assoc. (2019) 8:e013602. 10.1161/JAHA.119.013602 31495251 PMC6818019

[60] Yu X Li H Murphy TA Nuss Z Liles J Liles C. Angiotensin II type 1 receptor autoantibodies in postural tachycardia syndrome. J Am Heart Assoc. (2018) 7:e008351. 10.1161/JAHA.117.008351 29618472 PMC6015435

[61] Katz RM. Cough syncope in children with asthma. J Pediatr. (1970) 77:48–51. 10.1016/S0022-3476(70)80043-9 5430795

[62] Jain AM. Cough syncope. Indian J Pediatr. (1971) 38:434–6. 10.1007/BF02831369 5143566

[63] Charcot JM Statement to the societe de biologie. Gaz Med de Paris. (1876) 1876:588e9.

[64] Haslam RHA Freigang B. Cough syncope mimicking epilepsy in asthmatic children. Can J Neurol Sci. (1985) 12:45–7. 10.1017/S0317167100046576 3978474

[65] O'Doherty DS. Tussive syncope and its relation to epilepsy. Neurol. (1953) 3:16–21. 10.1212/WNL.3.1.16 13013494

[66] DeMaria AA Jr Westmoreland BF Sharbrough FW. EEG in cough syncope. Neurol. (1984) 34:371–4. 10.1212/WNL.34.3.371 6538280

[67] Mcintosh HD Estes EH Warren JV. The mechanism of cough syncope. Amer Heart Jour. (1956) 52:70–82. 10.1016/0002-8703(56)90119-3 13326836

[68] Waldmann V Combes N Narayanan K Sharifzadehgan A Bouzeman A Beganton F. Cough syncope. AM J Med. (2017) 130:e295–6. 10.1016/j.amjmed.2017.01.050 28238688

[69] Sharpey-Schafer EP. The mechanism of syncope after coughing. Br Med J. (1953) 2:860–3. 10.1136/bmj.2.4841.860 13094038 PMC2029864

[70] Benditt DG Samniah N Pham S Sakaguchi S Lu F Lurie KG. Effect of cough on heart rate and blood pressure in patients with “cough syncope”. Heart Rhythm. (2005) 2:807–13. 10.1016/j.hrthm.2005.04.022 16051114

